# Comprehensive characterization of the phosphoproteome of gastric cancer from endoscopic biopsy specimens

**DOI:** 10.7150/thno.37623

**Published:** 2020-01-12

**Authors:** Yuichi Abe, Hidekazu Hirano, Hirokazu Shoji, Asa Tada, Junko Isoyama, Akemi Kakudo, Daigo Gunji, Kazufumi Honda, Narikazu Boku, Jun Adachi, Takeshi Tomonaga

**Affiliations:** 1 Laboratory of Proteome Research, National Institute of Biomedical Innovation, Health and Nutrition, Ibaraki, Osaka 567-0085, Japan; 2 Laboratory of Proteomics for Drug Discovery, Center for Drug Design Research, National Institute of Biomedical Innovation, Health and Nutrition, Ibaraki, Osaka 567-0085, Japan; 3 Gastrointestinal Medical Oncology Division, National Cancer Center Hospital, Tsukiji, Chuo-ku, Tokyo, 104-0045, Japan; 4 Department of Biomarkers for Early Detection of Cancer, National Cancer Center Research Institute, Tokyo 104-0045, Japan

**Keywords:** Endoscopic biopsy, Phosphoproteomics, Precision medicine, Kinome

## Abstract

**Rationale**: Cancer phosphoproteomics can provide insights regarding kinases that can be targeted for therapeutic applications. Monitoring the phosphoproteomics in cancer is expected to play a key role in optimizing treatments with kinase inhibitors. Clinical phosphoproteomics in surgical tissues and patient-derived models has been studied intensively. However, the reported data may not accurately reflect the phosphosignaling status in patients due to the effect of ischemia occurring during surgery or changes in the characteristics of cancer cells when establishing the models. In contrast, endoscopic biopsies have an advantage for clinical phosphoproteomics because they can be rapidly cryo-preserved. We aimed to develop a highly sensitive method for phosphoproteomics in endoscopic biopsies of gastric cancer.

**Methods**: Three tumor biopsies and three normal gastric biopsies were obtained by endoscopy at one time, and subjected to our optimized phosphoproteomics. Phosphopeptides were enriched with an immobilized metal affinity chromatography, and labeled with Tandem Mass Tag reagent. Quantified phosphosites were compared between the pairs of tumor/normal biopsies within same patient. Cancer-specific activated pathways and kinases were identified by pathway enrichment analysis and kinase-substrate enrichment analysis.

**Results**: Our protocol enabled the identification of more than 10,000 class 1 phosphosites from endoscopic biopsies. A comparison between samples from cancer tissue and normal mucosa demonstrated differences in the phosphosignaling, including biomarkers of response to DNA damage. Finally, cancer-specific activation of DNA damage response signaling was validated by additional phosphoproteomics of other patients and western blotting of gastric cancer/normal cells.

**Conclusion**: In summary, our pioneering approach will facilitate more accurate clinical phosphoproteomics in endoscopic biopsies, which can be applied to monitor the activities of therapeutic kinases and, ultimately, can be a useful tool to precision medicine.

## Introduction

Precision medicine has attracted attention as a next-generation strategy in cancer therapy 1. It is expected to improve clinical outcomes by facilitating the optimal drug selection that will target patient-specific oncogenic characters 2. Recently, large-scale molecular profiling of clinical cancer tissue samples has been done via various genomics-based projects 3. Furthermore, the integration of genomic, epigenomic, transcriptomic, and proteomic data has been attempted in the context of precision medicine 4-6. Multi-omics profiling, especially genomic profiling, has been used to identify novel subtypes of cancer 7. However, there is a limitation in the genotype-based therapeutic strategies due to the differences between the oncological genotypes and phenotypes of various patients 8.

Phosphorylation, which is a critical regulator of protein function, has been reported to be associated with cancer-specific cellular phenotypes (called “cancer hallmarks”) 2. Therefore, in the field of cancer biology, various strategies have been developed to characterize onco-phosphosignaling 9. Genomic analysis has revealed many driver mutations in protein kinases, which are the enzymes that catalyze protein phosphorylation. However, monitoring kinase activity based on genomic and transcriptomic data is difficult because most genomic data do not directly reflect enzymatic activities 10. Recently, efforts to conduct clinical phospho-proteogenomic analyzes using surgical tissues of breast and ovarian cancer have shed light on characteristic subtypes and profiles of the active kinases in these types of cancers 5, 6. It is expected that comprehensive understanding of patient-specific abnormalities in phosphosignaling by phosphoproteomic analysis may improve the ability to predict treatment outcomes and select optimal kinase inhibitors 11.

To improve the clinical applicability of phosphoproteomics, we have developed techniques such as a selected reaction monitoring method for phosphorylated peptides 12, 13, a highly sensitive phosphotyrosine (pY)-immunoprecipitation technique 14, and a phosphoproteomic workflow using cultured cells and organoid cultures 15 16. So far, we conducted comparative profiling of the active kinases in Cetuximab-sensitive and Cetuximab-resistant cell lines 16. The results showed that phosphoproteomics could be used to predict drug sensitivity and, may be used as a tool in determining the optimal treatment for each patient. However, it is well known that there is a divergence of the molecular properties between cancer *in vivo* and patient-derived cancer models. Therefore, it is necessary to use clinical samples which directly represent the characteristics of patients' cancer tissues. If the phosphorylation status in cancer tissue can be measured by the phosphoproteomic analysis using a biopsy sample, the obtained kinome profiling (indicating activated kinases) data can be used to identify the most suitable strategy of treatment (e.g., a kinase inhibitor).

However, the artificial phosphoproteomic changes caused by ischemia during surgical procedures hamper the interpretation of the phosphoproteomic data as reported in previous projects 17. Endoscopic biopsy specimens from gastric cancer patients are preferred for clinical phosphoproteomic analysis due to less artificial phosphoproteomic change, because they can be kept fresh (it takes less than a minute to freeze them).

Currently, the application of conventional phosphoproteomics into clinical usage requires samples in large quantities (usually more than 1 mg). Thus, in this study, we conducted phosphoproteomics on endoscopic biopsies from patients with gastric cancer to comprehensively evaluate the phosphosignaling and potentially therapeutic kinases in *in vivo* condition. We could identify more than 10,000 class 1 phosphosites (localization probability, 0.75-1.00; median > 0.996). Then, we compared the phosphoproteomic profile between normal and cancer specimen pairs. As a result, cancer-specific activated pathways and kinases were identified by pathway enrichment analysis 18 and kinase-substrate enrichment analysis (KSEA) 19 from the obtained phosphoproteomic profiles.

## Methods

### Reagents and antibodies

A tandem mass tag (TMT) 10-plex isobaric label reagent set and Lithium dodecyl sulfate (LDS) sample buffer were purchased from Thermo Fisher Scientific (Waltham, MA). The PhosSTOP™ phosphatase inhibitor cocktail, cOmplete™ protease inhibitor cocktail, and Trypsin were obtained from Roche (Basel, Switzerland). Phosphate-buffered saline tablets were purchased from Takara (Shiga, Japan). A detergent-compatible (DC) protein assay kit was obtained from Bio-Rad (Hercules, CA). XV Pantera gradient gel (5 to 20%) was purchased from DRC (Tokyo, Japan). An ImmunoStar LD kit and Lysyl Endopeptidase (Lys-C) were obtained from Wako (Osaka, Japan). Oasis HLB cartridges were purchased from Waters (Milford, MA).

ATM (D2E2), pCHEK1 S345 (133D3), and pCHEK2 T68 (C13C1) antibodies were obtained from Cell Signaling Technology (Danvers, MA). CHEK1 (DCS-310) and CHEK2 (DCS-270) antibodies were obtained from MBL (Nagoya, Japan). pATM S1981 (10H11.E12) antibody was purchased from Merck (Burlington, MA). ATR (N-19) antibody was obtained from SantaCruz (Dallas, TX). α-Tubulin (DM1A) antibody was purchased from Thermo Fisher Scientific.

### Patient characteristics

Five patients that were diagnosed with advanced gastric cancer were recruited for participation in this study; All the patients had primary tumors and received endoscopic procedures to collect biopsy specimens from those tumors. Three patients (60%) had diffuse-type histology according to the Lauren classification. One patient (20%) had HER2-positive gastric cancer (3+ as assessed by immunohistochemistry [IHC]) while the other four patients (80%) had HER2-negative gastric cancer (0 or 1+ as assessed by IHC). Two patients (40%), including the patient with HER2-positive gastric cancer and one patient with HER2-negative gastric cancer, had not received chemotherapy before the endoscopic biopsy procedures while three patients (60%) underwent the endoscopic biopsy procedures after being treated with chemotherapies including S-1 monotherapy, S-1 plus cisplatin, and S-1 plus oxaliplatin.

For validation of the phosphoproteomic experiments, four patients that were diagnosed with advanced gastric cancer were recruited for participation. All the patients had primary tumors and received endoscopic procedures to collect biopsy specimens from their tumors. Two patients (50%) had diffuse-type histology according to the Lauren classification. Two patients (50%) had HER2-positive gastric cancer, whereas the other patients (50%) had HER2-negative gastric cancer. All patients had not received chemotherapy before the endoscopic biopsy procedures.

### Collection of endoscopic biopsies in gastric cancer

The Institutional Review Board of the National Cancer Center Hospital (Tokyo, Japan) and the National Institute of Biomedical Innovation, Health, and Nutrition (Osaka, Japan) approved the collection and analyses of the endoscopic samples. All the patients were treated at National Cancer Center Hospital. Written informed consent was obtained from all the subjects. Three tumor biopsies and three normal gastric biopsies were obtained by endoscopy at one time. After collection, each sample was placed in a separate screw-cap tube and immediately (within 20 seconds) snap-frozen in liquid nitrogen. The frozen samples were stored at -80°C until the subsequent sample preparation.

### Homogenization and solubilization of endoscopic biopsy tissues

Each frozen sample was transferred to a 1.5-mL tube and mixed with phase transfer surfactant (PTS) buffer (50 mM ammonium bicarbonate, 12 mM sodium deoxycholate, 12 mM sodium lauroyl sarcosinate) supplemented with cOmplete™ protease inhibitor and PhosStop™ phosphatase inhibitor 20. Each biopsy was homogenized in a PowerMasher 2 (Nippi, Tokyo, Japan) for 30 seconds and subsequently boiled at 95°C for 5 min. The lysates were further sonicated three times (15 min per cycle) with a Bioruptor sonicator (Cosmo Bio, Tokyo, Japan). After sonication, the protein concentration was measured with DC protein assay kit.

### Protein precipitation and digestion

To remove contaminants, the cell lysate to have 500 μg of protein was precipitated by methanol/chloroform precipitation. The pellets were re-suspended in PTS buffer and the amount of protein in the re-suspended solution was measured with the DC protein assay kit. Based on the measured protein concentration, a volume containing 320 μg of protein lysate was subjected to reduction by dithiothreitol (DTT) and alkylation by iodoacetamide (IAA). In the second phosphoproteomics, 300 μg of protein lysate per each TMT channel was used for the following experiment. Reduction was performed by incubation with 10 mM DTT for 30 min at RT. Alkylation was conducted by incubation with 25 mM IAA for 30 min at RT in the dark. Then, the samples were diluted four times in 50 mM ammonium hydrogen carbonate, mixed with trypsin (protein weight, 1/50) and Lys-C (protein weight, 1/50), and subjected to incubation for 18 h at 37°C.

### Desalting and enrichment of phosphopeptides

Desalting of the peptides was conducted with OASIS HLB as described previously 14. Enrichment of the phosphopeptides was done with Fe^3+^ IMAC resin based on the protocol described previously 21. Here the enrichment was carried out on a Fe-IMAC/C18 StageTip: the C18 disc was set in a 200 μl disposable tip, the Fe-IMAC resin was set on the C18 disc, and the desalted peptides were passed through the IMAC/C18 StageTip. The phosphopeptides were eluted with 1% H_3_PO_4_ and purified on the C18 disc.

### TMT labeling and fractionation of phosphopeptides on C18-SCX StageTip

To quantify phosphosites in each biopsy, TMT labeling was conducted on each phosphopeptide-enriched sample according to the manufacturer's protocol. Labeled phosphopeptides were fractionated into seven fractions using the C18-SCX StageTip in pH/salt step gradient mode as described previously 22. The elution buffer components of the seven fractions were as follows: the first fraction included 0.5% TFA, the second fraction included 1% TFA, the third fraction included 2% TFA, the fourth fraction included 3% TFA, the fifth fraction included 100 mM ammonium acetate and 3% TFA, the sixth fraction included 500 mM ammonium acetate and 4% TFA, and the seventh fraction included 500 mM ammonium acetate.

### LC-MS/MS analysis

The phosphoproteomic analysis was conducted with Q Exactive Plus (Thermo Fisher Scientific, Waltham, MA, USA) coupled with Ultimate 3000 (Thermo Fisher Scientific) and an HTC-PAL (CTC Analytics, Zwingen, Switzerland). The nano-liquid chromatography gradient was formed of Buffer A (0.1% formic acid and 2% acetonitrile) with 5-30% Buffer B (0.1% formic acid and 90% acetonitrile) over 135 minutes; the flow rate was 280 nL/min. The settings used for the Q Exactive Plus were similar to those described in a previous phosphoproteomic study 14.

### Phosphopeptide identification and quantification

Phosphopeptide identification was carried out with MaxQuant 1.5.1.2 supported by the Andromeda search engine 23. The UniProt database for human proteins (release 2017_01) combined with 262 common contaminants was used to analyze the LC-MS/MS data. The enzyme specificity was set to trypsin/P (the C-terminal of Arg or Lys with cleavage at the proline bond allowed). Instances of incorrect cleavage of up to two sites were tolerated. A fixed modification of carbamidomethylation of cysteine residue was assumed while methionine oxidation and serine, threonine, and tyrosine phosphorylation were set to variable modifications. The false discovery rate (FDR) of protein groups, peptides, and phosphosites were less than 0.01. Quantitative values of the phosphorylation sites across different fractions were automatically integrated and summarized in “Phospho (STY) Sites.txt” by MaxQuant. Peptides that were identified from reversed database, or identified as potential contaminants were not used in the following analysis. Other parameters used for MaxQuant were summarized in Table S5. The cut-off criterion for the localization probability at each phosphosite was more than 0.75 24.

### Data processing and visualization

The statistical analysis was carried out with Perseus 1.6.0.2 (www.perseus-framework.org) 25. The quantitative TMT reporter ion intensities were log_2_ transformed and normalized by median centering of the values in each TMT channel. Phosphosites with quantitative values in at least one of the 10 TMT channels were used for subsequent statistical analysis. Imputation of the missing value was not conducted. A two-tailed Welch *t*-test was conducted to check determine if the differences between the phosphosite values for in normal and cancer tissues were statistically significant. A permutation test was performed to calculate the adjusted *q* values. Based on the fold change and *p* value, significant differences for the phosphosites were determined in the significance analysis of microarray (SAM), as reported previously 26. In the SAM, we applied the following parameters: S0 = 0.1, FDR < 0.05. Phosphosites with statistical significances were summarized in Table S2. Proportional Venn diagrams were prepared using eulerAPE 27. The correlation matrix was constructed using the R package called “Correlation Matrix.” Perseus software was used to create the principle component analysis (PCA) plots.

### Pathway analysis and kinome profiling

The class 1 phosphosites exhibiting statistically significant differences between healthy and cancer tissues were subjected to a pathway analysis in WebGestalt 28. Two pathway analyses were conducted by using pathway information in KEGG 29 and that in WikiPathway 30.

Kinome profiling was conducted using the phosphoproteomic data as described previously 16. The kinase activity was predicted using the KSEA algorithm 19 in the KSEA app 31, and the PTM-Signature Enrichment Analysis (SEA) algorithm 18. Results of KSEA with more than three kinase-substrate relationships downloaded from PhosphositePlus (shown as “m” in Table S4) and *p* values less than 0.05 was used in this study. In the PTM-SEA, kinases with *p* values less than 0.05 were used.

### Western blotting to validate the expression and phosphorylation status of the kinases in DDR signaling and ERBB2 activity

A 5 to 20% XV Pantera gradient gel was used for sodium dodecyl sulfate polyacrylamide gel electrophoresis. The cell lysate in PTS buffer was mixed with the LDS sample buffer and boiled at 95°C for 5 min. The cell lysates were subjected to electrophoresis at 120 V for 35 min. Proteins were blotted to polyvinylidene difluoride (PVDF) membranes at 40 V for 70 min. The PVDF membranes were incubated at RT in specific antibodies and horse radish peroxidase (HRP)-conjugated antibodies for detection. The incubation times with specific antibodies and HRP-conjugated antibodies were 18 hours and 1 hour, respectively. Chemiluminescent measurements of the blotted proteins were performed using ImmunoStar with LAS 4000 (GE Healthcare, Chicago, IL).

## Results

### Comprehensive phosphoproteomic analysis of endoscopic biopsies

**Figure 1:**
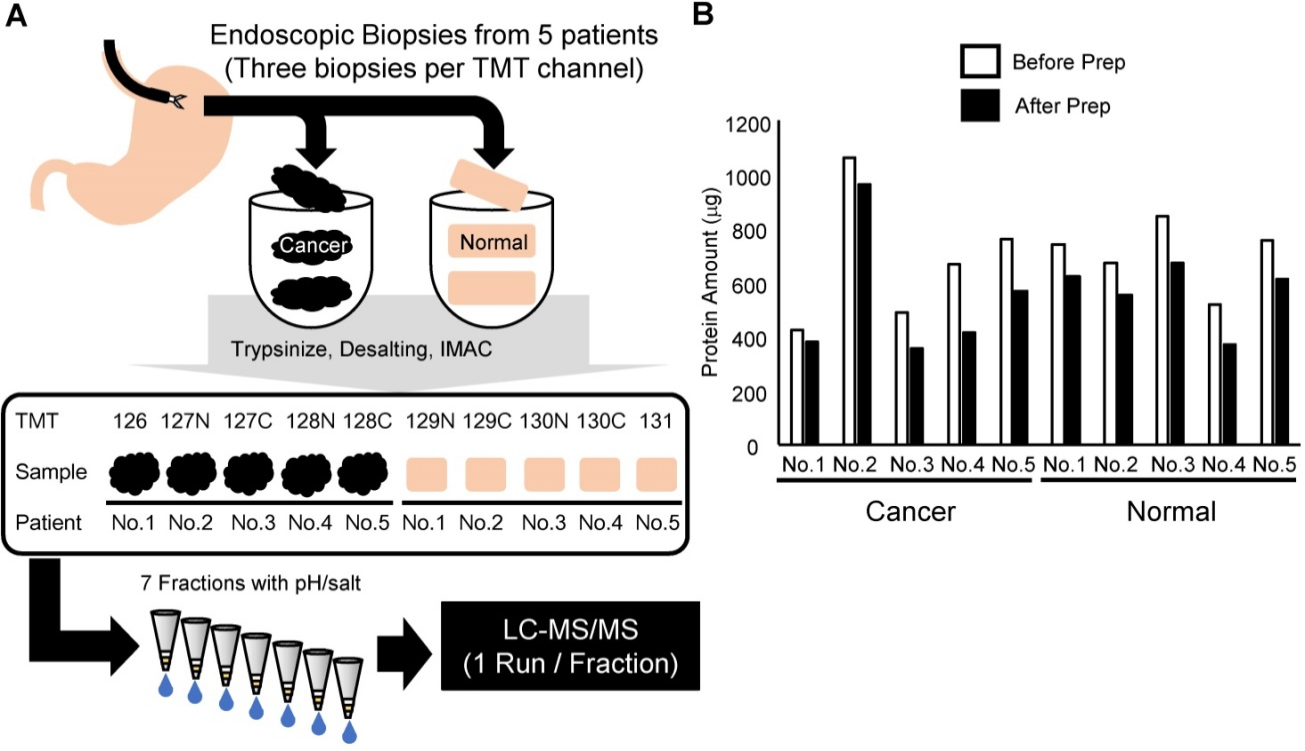
Summary of the experimental approach in this study: A. Workflow of the phosphoproteomic analysis from endoscopic biopsies. B. Amount of protein lysate before and after methanol/chloroform precipitation in each sample collected.

In this study, we developed a phosphoproteomic procedure using three pieces of endoscopic biopsies for each TMT channel (Figure 1A). Clinical information on the patients is summarized in Table 1. The protein lysate was extracted and analyzed from the obtained endoscopic biopsies. A primary objective was to reduce the sample loss during the procedure. However, tissue samples contain large amounts of contaminants, which hamper the sensitivity of liquid chromatography-tandem mass spectrometry (LC-MS/MS). Thus, methanol/chloroform precipitation was included before protein digestion in the protocol. Moreover, to minimize the loss of the small amount of phosphopeptides in the digested biopsy sample, we performed enrichment of phosphopeptides with an immobilized metal affinity chromatography (IMAC) on C18 StageTip 32. To improve depth of phosphoproteomics without loss of samples, phosphopeptides were further fractionated with C18-Strong Cation Exchange (SCX) StageTip 22 instead of conventional fractionation of peptides with off-line LC. The amount of protein in each mixture of three endoscopic biopsies ranged from 350 to nearly 1,000 μg, indicating that at least 100 μg of protein lysates could be obtained consistently from each biopsy (Figure 1B). After the methanol/chloroform precipitation, 62 to 90% of the protein was recovered relative to the amount before precipitation (Figure 1B). Because the smallest sample was 320 μg, the same amount of protein (320 μg) among 10 samples was subjected to further phosphoproteomic analysis.

### Phosphoproteomic status in the endoscopic biopsies

**Figure 2:**
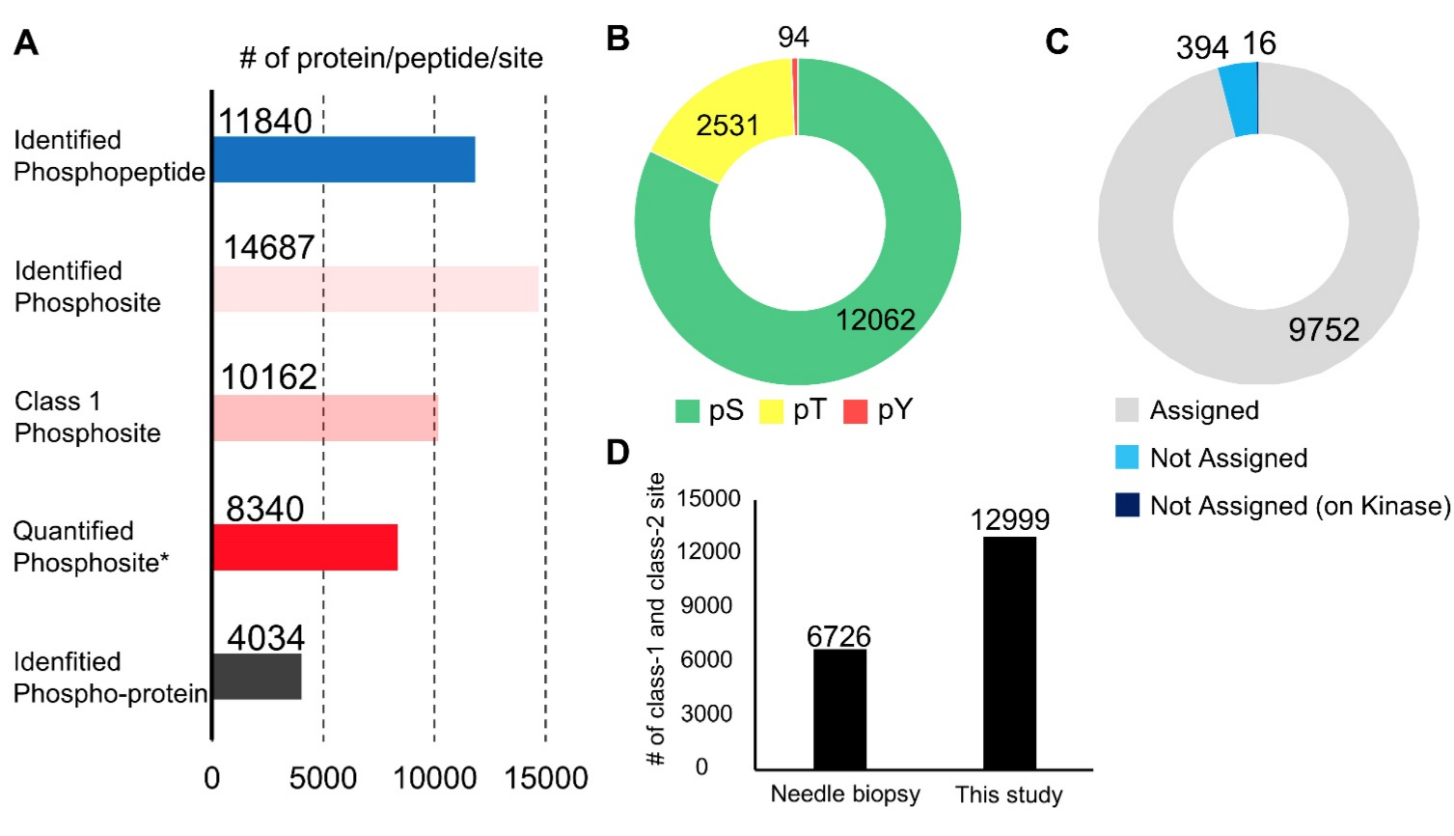
Summary of the phosphoproteomic results from endoscopic biospsies: A. Number of phosphopeptides, phosphosites, class 1 phosphosites, class 1 phosphosites with quantitation, and phospho-protein groups. Asterisk (Quantified phosphosites) means the phosphosites with quantitative values in at least one channel of TMT 10-plex labels; B. The proportions of phosphoserine (green), phosphothreonine (yellow), phosphotyrosine (red); C. The proportions of class 1 phosphosites that were assigned in the PhosphositePlus database (gray), those not assigned (light blue), and those on kinases among phosphosites without assignment in the PhosphositePlus (dark blue); and D. A comparison of the numbers of class 1 and class 2 phosphosites identified in the previous study and those identified in the present study.

In the phosphoproteomic analysis of the endoscopic biopsy samples, 11,840 phosphopeptides, 14,687 phosphosites, 10,162 class 1 phosphosites, and 4,034 phospho-proteins were identified (Figure 2A and Table S1). Among the class 1 phosphosites, 8,340 phosphosites were quantified in at least one channel of a TMT 10-plex label (Figure 2A). All identified peptides and class 1 phosphosites are summarized in Table S1. The 14,687 phosphosites included 12,062 phosphoserine sites (82.1%), 2,531 phosphothreonine sites (17.2%), and 94 pY sites (0.7%) (Figure 2B). In addition, 394 (3.9%) of the detected 10,162 class 1 phosphosites are not assigned in the PhosphositePlus database 33, and 16 phosphosites which were localized on protein kinases were also not assigned in the database (Figure 2C). This implies that the proposed procedure is highly sensitive for identification of many unknown phosphosites, including potentially regulatory phosphosites on kinases.

This data was further compared with that of a previously reported phosphoproteomic study using biopsies collected with needle 34. In the previous study, needle biopsies from hepatocellular carcinoma were used for phosphoproteomics using Super-SILAC protocol 34. They performed 4 phosphoproteome analyzes with 0.17 mg to 1.4 mg protein lysate and identified 6,726 class 1 and class 2 phosphosites in total. On the other hand, our protocol made it possible to identify as many as 12,999 class 1 and class 2 phosphosites as nearly twice as in the previous results using needle biopsies (Figure 2D). Although the cancer types, the sample amounts, and the analytical platform were different between the previous and current study, these results indicate our optimized protocol is outstanding for obtaining global phosphoproteome data with a depth of 10K phosphosite identification from a small amount of clinical specimens, such as endoscopic biopsies.

### Quantitative signatures of cancerous and normal regions in subjects with gastric cancer

To check whether endoscopic biopsies reflect the oncogenic phosphosignaling in cancer cells in the tissue, a quantitative analysis of the phosphoproteomic data was performed. A total of 8,340 phosphosites with quantitative values in at least one TMT channel were subjected to the following quantitative analysis, including pathway enrichment analysis and kinome profiling.

**Figure 3:**
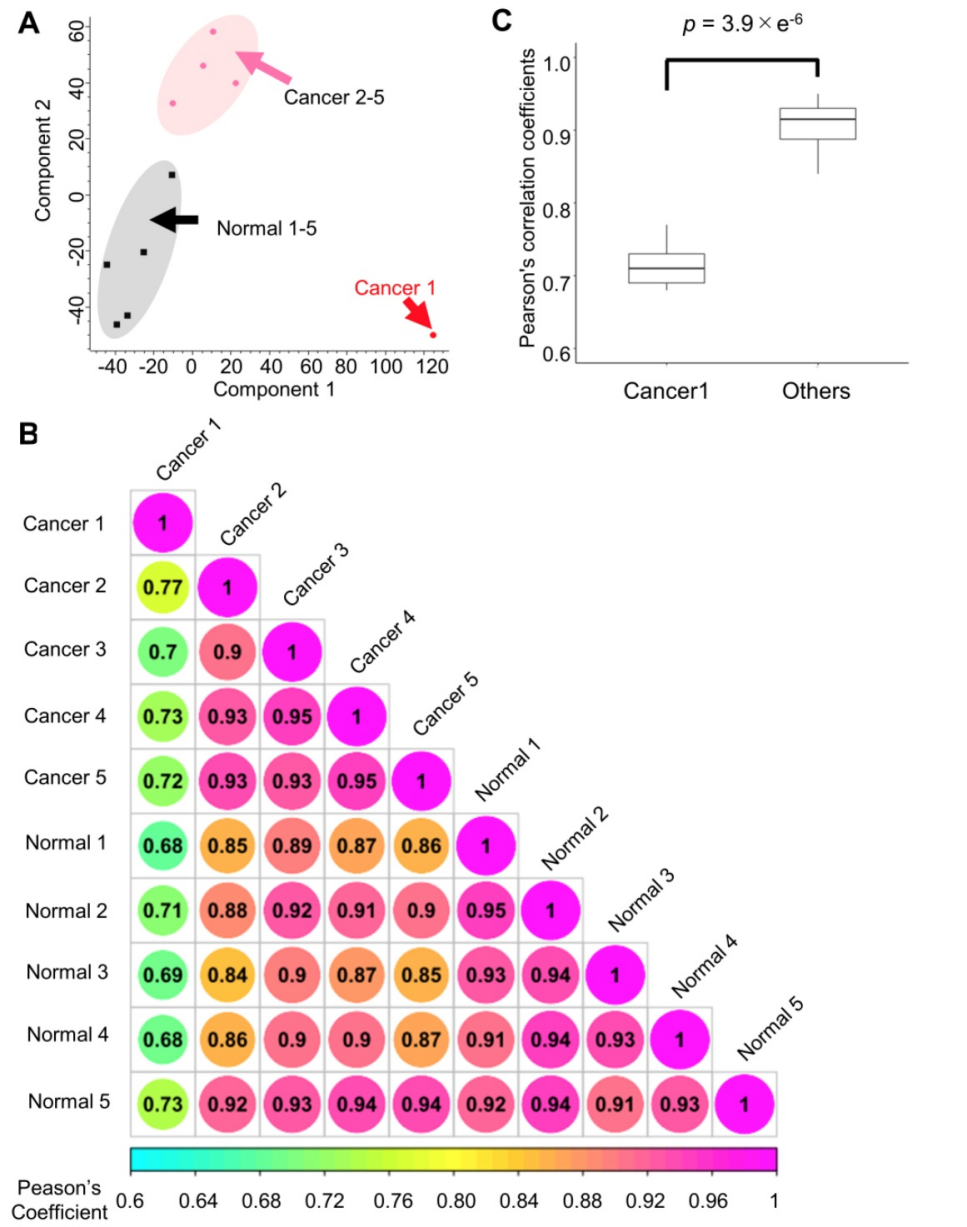
Quantitative results from the phosphoproteome analysis of endoscopic biopsy samples: A. Principle component analysis of the phosphoproteome data from cancer tissues (sample from subject no. 1: red; other samples: pink) and those from normal tissues (black) Normalized intensities in each channel were used for the analysis; B. Correlation matrix of the phosphoproteomic data; and C. Comparison of the Pearson's correlation coefficients with and without the “Cancer 1” sample from subject no. 1. Mann-Whitney U test was conducted to confirm the statistical difference.

First, the quantitative signature of the phosphoproteomic data in each endoscopic biopsy sample was obtained. Then, PCA was done to clearly classify the phosphoproteomic results of the normal tissues (gray area) and cancer tissues (magenta area). However, in this analysis, the data for the cancer tissue collected from subject no. 1 exhibited notably different characteristics compared with the other cancer biopsies (Figure 3A). A correlation analysis revealed that the Pearson's correlation parameters of the cancer tissue from subject no. 1 with all other samples (0.68 to 0.77, *p*-value = 3.9 × 10^-6^) were significantly lower than those of other pairs of samples (Figure 3B, 3C). This finding confirmed that cancer tissue from subject no.1 was quite different from any of other data points (Figure 3B). The reason for this inconsistency is unknown; however, to avoid unnecessary bias, the matched pair of cancer and normal tissues from the subject no.1 was excluded in the subsequent pathway analysis.

**Figure 4:**
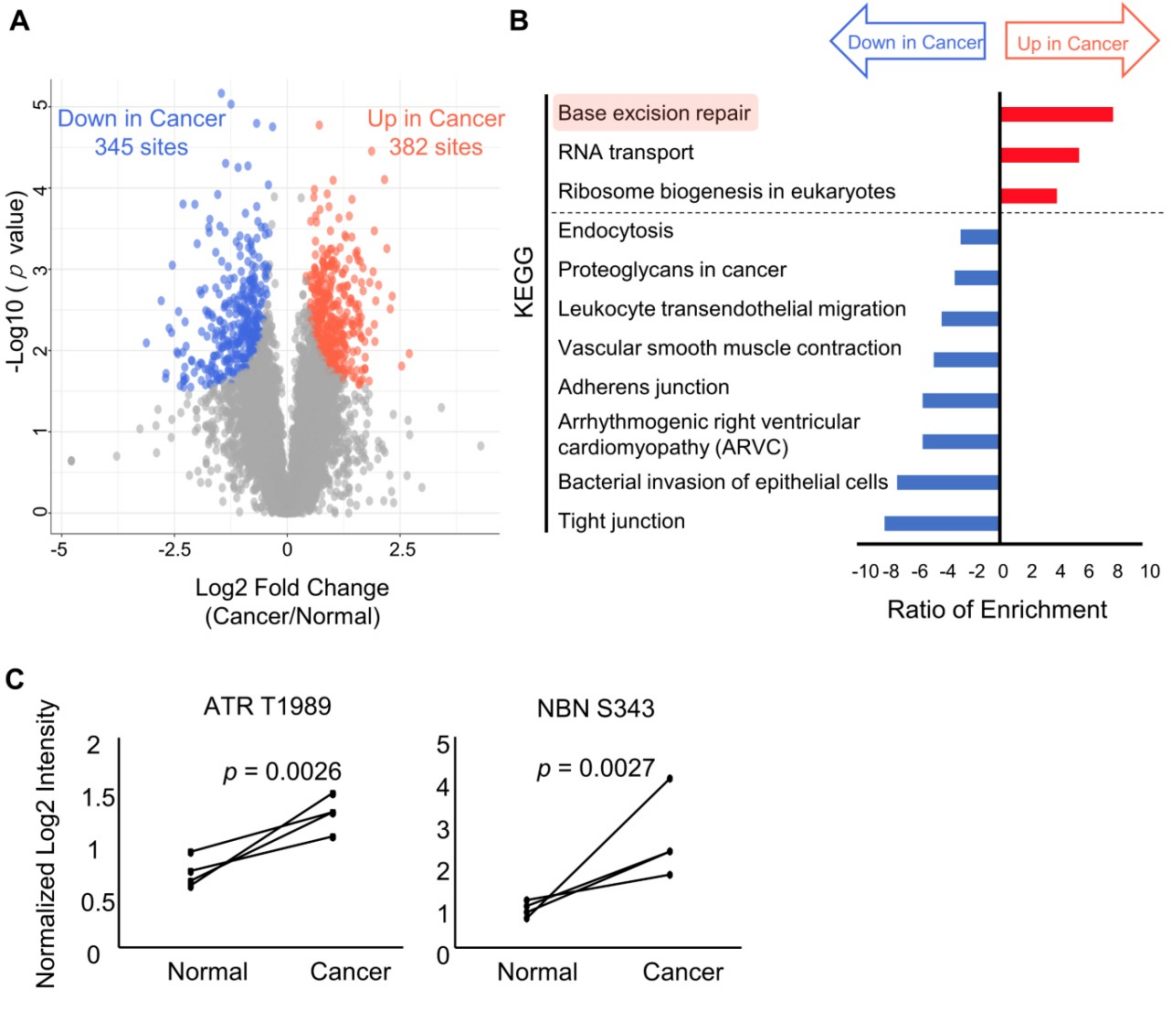
Results of the pathway analysis for comparing the phosphoproteomic data from cancer tissues and normal tissues: A. A volcano plot of the log_2_ fold change in each phosphosite and the log_10_ of the *p* values. Phosphosites with significant increases and decreases in the cancer tissues compared to the normal tissues show red and blue circles, respectively. Phosphosites without statistically significant differences show gray circles. B. A pathway analysis based on the pathway information in KEGG showing pathways that have *p* value less than 0.01 representing enrichment in the phosphosites that are increased in cancer tissues relative to normal tissues (red bars) and enrichment in the phosphosites that are decreased in cancer biopsies (blue bars); C. Paired dot plots of the normalized intensities of two phosphosites (ATR T1989 at left and NBN S343 at right) with the pairs of cancer and normal tissues from same patient connected by solid black lines.

Next, the phosphosites that exhibited significant differences between cancer and normal tissues were selected. A Welch t-test adjusted by permutation test was used to identify 382 phosphosites that were increased in cancer tissues (magenta circles) and 345 phosphosites which were decreased in cancer tissues (cyan circles), as shown in Figure 4A and Table S2. Those phosphosites were subjected to pathway analyses by WebGestalt 28 using different databases (KEGG 29 and WikiPathway 30). Results of pathway analysis with *p* values less than 0.01 are summarized in Table S3. Comparing the results obtained from the pathway analyses with the two databases, many modulations that are commonly considered to be “cancer hallmarks” 2 were identified in both (Table S3). Among the increased pathways in cancer, several pathways involved in genome instability and mutation, which is one hallmark of cancer, were identified based on both KEGG (Figure 4B, magenta) and WikiPathway (Figure S1, magenta). Moreover, base excision repair was significantly enhanced based on the phosphosites that were upregulated in cancer according to the KEGG database. Similarly, six pathways related to the response to DNA damage were significantly enriched according to the WikiPathway database.

The activation of ATR, a regulator of DNA damage response (DDR), was confirmed by the observation of increased phosphorylation of the 1989 serine residue of ATR (Figure 4C), which has been reported to be involved in regulating enzymatic activity of ATR 35. Furthermore, the NBN 343 serine residue, a known substrate of ATR 36, was also highly phosphorylated in cancer specimens, indicating that the signaling cascade via ATR was activated (Figure 4C). Therefore, our phosphoproteomic data on endoscopic biopsies demonstrated cancer-specific activation of DDR signaling in gastric cancer.

### Cancer-specific kinome profiling from phosphoproteomics on endoscopic biopsies

**Figure 5:**
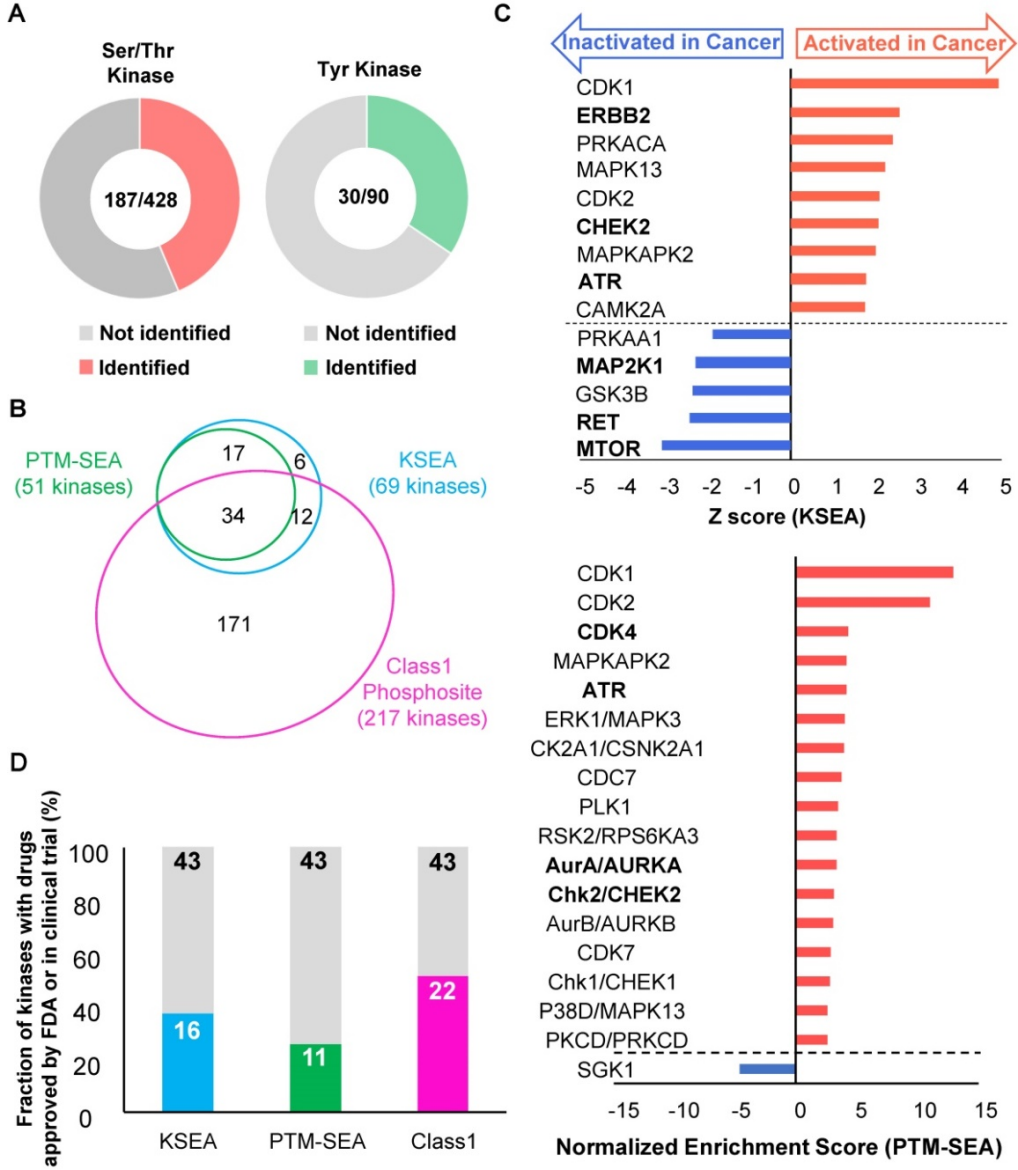
Kinome profiling using the phosphoproteomic data: A. The number of Ser/Thr kinase (left chart, red) and Tyr kinase (right chart, green) whose class1 phosphosites on the kinases themselves were identified is shown. B. A proportional venn diagram of the three kinomes identified from KSEA (Cyan), PTM-SEA (Green), and class 1 phosphosite on kinase (Magenda) is shown. C. Kinome profiling is shown to compare the phosphoproteomic data of the cancer tissues and normal mucosas. Kinases with a *p* value < 0.05 based on the KSEA (upper) and the PTM-SEA (lower) algorithm are shown. These kinases are predicted to be activated (red bars) and inactivated (blue bars) in cancer tissue. Kinases whose drugs are approved by FDA-approved or in clinical trials are shown in bold font. D. Percentage of kinases whose drugs were approved by FDA or in clinical trials were plotted as cyan (identified by KSEA), green (identified by PTM-SEA), and magenta (identified as class 1 phosphosites on kinases), respectively.

Profiling of druggable active kinases can be useful to guide anti-cancer therapy on the basis of phosphoproteomic data obtained from endoscopic biopsy samples. Thus, kinome profiling was conducted according to the method reported in our previous study 16. First, the number of phosphosites that are on protein kinases was identified from the phosphoproteomic data. The results showed that phosphosites were detected for 187 out of 428 serine/threonine kinases and 30 out of 90 tyrosine kinases (Figure 5A). This finding implies that this phosphoproteomic procedure provides the ability to monitor the activities of many kinases on the basis of information on the regulatory phosphosites such as phosphorylation in kinase domain 37. Next, the phosphoproteomic data was examined to assess the activities of the kinases based on two phosphosite-based enrichment analyses, KSEA and PTM-SEA, by using kinase-substrate relationships 18, 19. KSEA and PTM-SEA predicted that activities of 69 and 51 kinases were modulated, respectively. In total, activity profiles of 240 kinases among the 518 kinome in the human genome were obtained during this analysis (Figure 5B). The list and overlap of kinases between the results of the three methods (class 1 phosphosites, KSEA, and PTM-SEA) for the first phosphoproteomics are summarized in Table S4. Among the kinases with KSEA and PTM-SEA scores, the activities of 14 and 18 of these were predicted to be significantly different in the cancer tissues compared to in normal tissues (Figure 5C, and Table S4). In the activated kinases, ERBB2, CHEK2, ATR, CDK4, and AURKA (Bold font in Figure 5C) are therapeutic targets and drugs approved by Food and Drug Administration (FDA) or in clinical trials 10. Notably, ERBB2 is a synonym of Her2, which is targeted as an antibody (Trastuzumab) that has been approved for treatment against gastric cancer. Furthermore, we examined how many kinases in our kinome profiling are listed among the 43 kinases with inhibitors or antibody drugs approved by the FDA or in clinical trials 10. We found that 16 kinases identified by KSEA (Figure 5D, cyan), 11 kinases identified by PTM-SEA (Figure 5D, green) and 22 kinases identified by the profile of class1 phosphosites on kinase (Figure 5D, magenta) are included in the above list of 43 kinases. These results suggest that kinome profiling has the potential to monitor activities of druggable kinases and may be able to apply drug repositioning of kinase inhibitors based on clinical phosphoproteomics of small biopsies not only in gastric cancer but also in other cancers 11.

In addition, CHEK2 and ATR are known to regulate DNA damage signaling. The cancer-specific activations of CHEK2 and ATR correspond to the results of the two pathway analyses (Figure 4B and Figure S1) and to the previous study, which demonstrated the functional importance of DDR signaling in gastric cancer cases 38. Although the CHEK2 inhibitor and ATR inhibitor are not currently approved for gastric cancer, previous studies reported that proliferations of gastric cancer cell lines were suppressed by a CHEK inhibitor 39 and an ATR inhibitor 40. Further validation of the pharmacological mechanism of those inhibitors would help us to understand the draggability of DDR signaling in gastric cancer.

**Figure 6:**
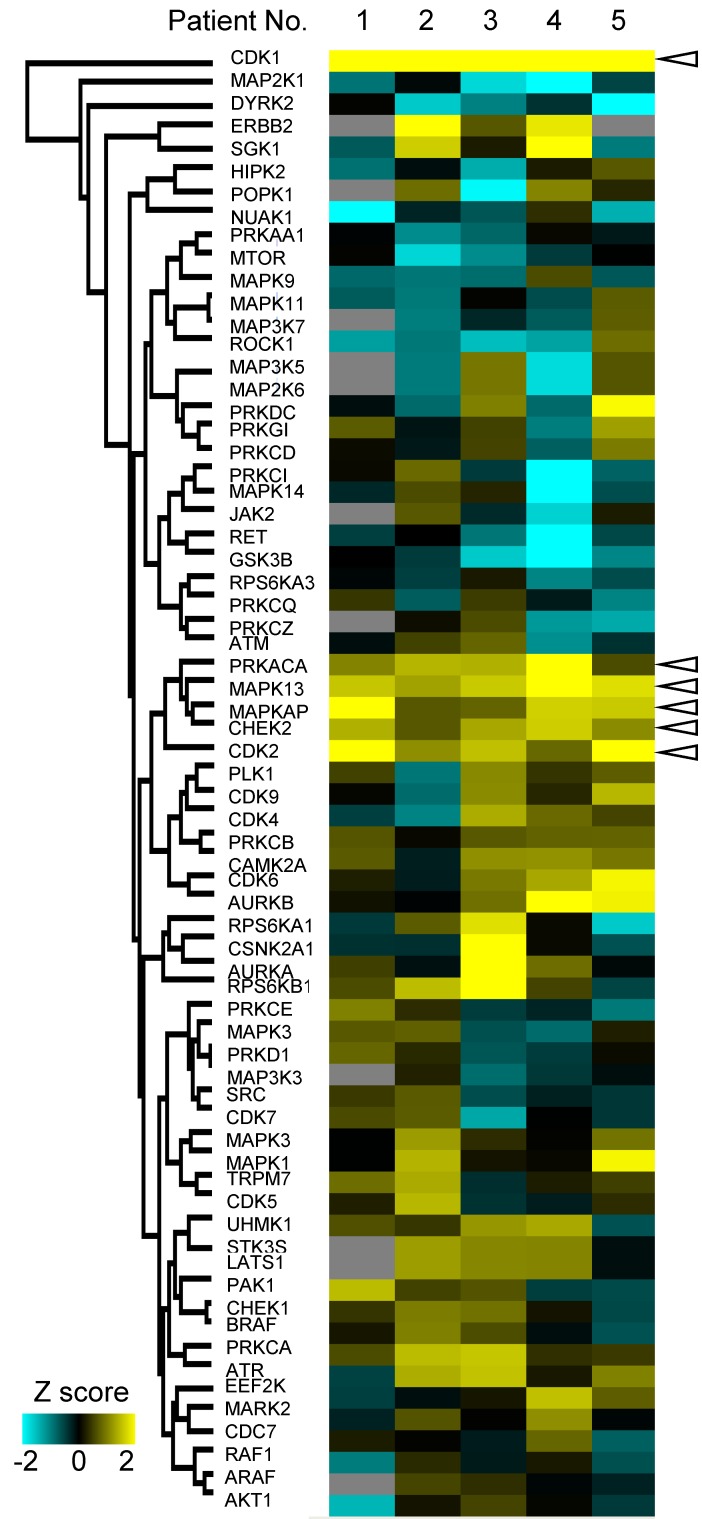
Personalized kinome profiling using the phosphoproteomic data in each patient: A heatmap of personalized kinome profiling with *Z* scores calculared by KSEA algolism. Yellow shows cancer-specific activation of each kinase. Cyan means inactivation of each kinase in cancer tissue compared with normal mucosas. Gray indicates missing values. Blanked arrowheads indicate commonly activated kinases in cancer tissues (CDK1, PRKACA, MAPK13, MAPKAP, CHEK2, and CDK2).

Furthermore, we examined variations of kinase activities in cancer between each patient. Some of kinases such as CDK1 and CHEK2 (blanked arrowheads in Figure 6) were commonly activated in cancer tissues among all patients, but activities of most kinases varied from patient to patient (Figure 6).

### Validation of cancer-specific activation of DDR signaling in gastric cancer

**Figure 7:**
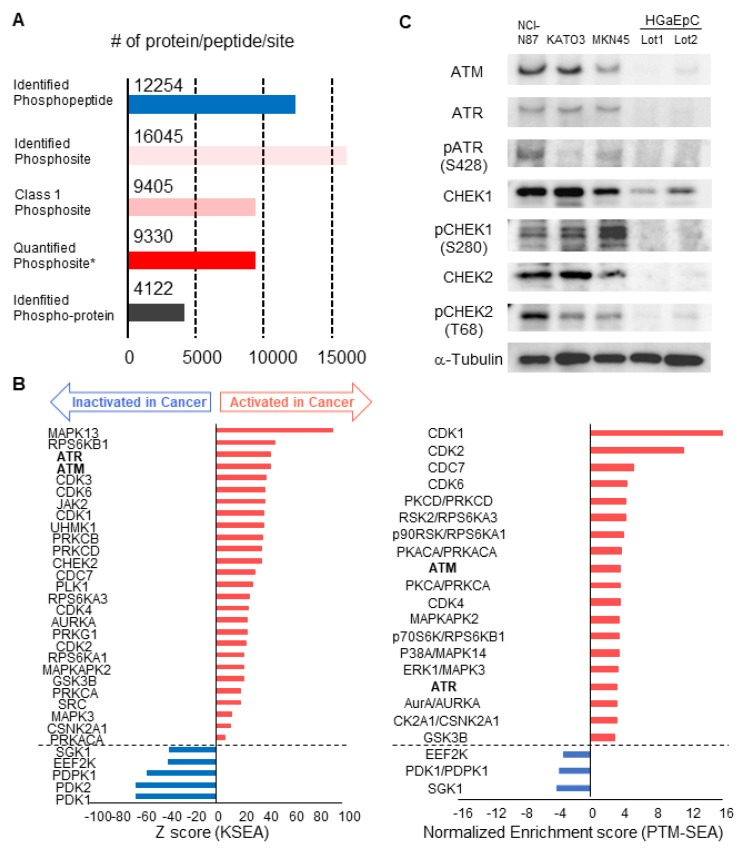
Validation of the cancer-specific activation of DDR signaling with phosphoproteomics from other patients and western blotting of gastric cancer/normal cells: A. Number of phosphopeptides, phosphosites, class 1 phosphosites, class 1 phosphosites with quantitation, and phospho-protein groups are shown. The asterisk (quantified phosphosites) represents the phosphosites with quantitative values in at least one channel of TMT 10-plex labels. B. Kinome profiling from the phosphoproteomic data of cancer tissues and normal mucosas is shown. Kinases with statistically significant differences are shown (*p* value < 0.05 based on the KSEA and PTM-SEA algorithm). These kinases are predicted to be activated (red bars) and inactivated (blue bars) in cancer tissue. ATM and ATR are shown in bold font. C. Expression and activity of kinases in DDR signaling were verified with western blotting. Three gastric cancer cell lines (NCI-H87, KATO3, and MKN45) were compared with two different series of HGaEpC and normal gastric epithelial cells. α-tubulin was used as an internal control.

Finally, we validated cancer-specific activation of DDR signaling with phosphoproteomics of endoscopic biopsies from other patients and western blotting of gastric cancer/normal cell lines. By using four pairs of endoscopic biopsies from cancer regions and from normal mucosa, an additional phosphoproteomics was conducted via the same protocol as shown in Figure 1. In the phosphoproteomic analysis, 12,254 phosphopeptides, 16,045 phosphosites, 9,405 class1 phosphosites, and 4,122 phospho-proteins were identified (Figure 7A, Table S6). The extent of phosphoproteomics in the second experiment was almost the same as in the initial analysis shown in Figure 2A. Next, we selected 361 and 388 phosphosites with statistical significances that were increased or decreased in the cancer region, respectively (Table S7). Those phosphosites were applied to the pathway analysis and to the kinome profiling in the same way as the initial phosphoproteomic data. The cancer-specific activation of DDR signaling was also shown during the pathway analysis of the second phosphoproteomic data (Figure S2 and S3, bold). The kinome profiling with KSEA and PTM-SEA further indicated cancer-specific activation of kinases in DDR signaling, such as ATM and ATR (Figure 7B, bold font), which agreed to the results of the initial phosphoproteomic analysis.

Furthermore, we compared the expression and phosphorylation status of the kinases in DDR signaling between gastric cancer/normal cells. We examined the expression of four kinases (ATM, ATR, CHEK1 and CHEK2) and three regulatory phosphosites on the kinases (ATR S428, CHEK1 S280, and CHEK2 T68) with western blotting. The results of KSEA and PTM-SEA in the second phosphoproteomics are summarized in Table S8. All of the kinases and the phosphosites were increased in the gastric cancer cell lines, NCI-N87, KATO-3, and MKN45, relative to the normal gastric cells (Figure 7C).

Taken together, cancer-specific activation of DDR signaling was validated by the second cohort experiment in addition to the experiments using the cancer cell lines. Thus, our phosphoproteomic procedure for endoscopic biopsy can accurately monitor modulation of phosphosignaling in cancer cells.

## Discussion

In this study, we examined the phosphoproteomics of cancer and normal tissues collected by endoscopic biopsy. The proposed analysis identified more than 10,000 class 1 phosphosites from only 320 μg of protein lysate per sample. The data revealed not only oncogenic characteristics that are well known but also several kinases that are activated in gastric cancer tissues that might be novel therapeutic targets. These results demonstrate the possibility of using endoscopic biopsies in precision medicine for determining the optimal anti-cancer drug based on a patient's comprehensive phosphoproteomic profile (specifically, the phosphorylation status of the collected cancer tissue).

It should be emphasized that comprehensive phosphoproteomics can be conducted on endoscopic specimens (Figure 2A). The proposed approach is expected to have a great influence on further clinical applications because biopsy samples can be quickly preserved in about ten seconds after resection to allow accurate phosphoproteomic data to be collected without the artificial phosphoproteomic changes caused by ischemia during surgical procedures. The kinome profiling in this study revealed that KSEA and PTM-SEA could be used to identify activation of druggable kinases in DDR signaling such as CHEK2 and ATR (Figure 5C), which is consistent with a previous report 38. The kinome profiling from phosphoproteomics of endoscopic biopsies also revealed cancer-specific activation or inactivation of several kinases whose inhibitors are approved by FDA or in clinical trials in other cancers (Figure 5D). Among the 43 kinases with clinical inhibitors, activities of 16 and 11 kinases were predicted from the KSEA and PTM-SEA, respectively (Figure 5C). These findings suggest that KSEA and PTM-SEA are suitable methods for screening drug targets by using phosphoproteomics. Additionally, comprehensive kinome profiling from phosphoproteomic data of endoscopic biopsy can provide information about previously unknown kinases that are activated in cancer tissues and, may be targeted for novel anti-cancer therapies.

Furthermore, endoscopic biopsies are the only means of obtaining tissue specimens repeatedly from patients with unresectable advanced gastric cancer; therefore, the proposed technique would make it possible to easily conduct global phosphoproteomics in many patients with unresectable cancer. Given that the biological behavior of tumor cells can change moment to moment, leading to resistance in response to selective pressures associated with cancer therapies, there is a need for real-time monitoring system in phosphorylation status for cancer patients receiving cancer therapies like kinase inhibitors. The real-time monitoring of phosphoproteomic of cancer *in vivo* would contribute to measure how the cancer tissue responds to an administered drug and allow rapid prediction of the next therapeutic target in case the cancer develops resistance to the drug. Furthermore, some phosphosignalings have been reported to be relevant to the efficacy of cancer immunotherapy 41. Thus, if it is combined with other omics data, our phosphoproteomic analysis may be useful for predicting sensitivity, not only to kinase inhibitors, but also to immune checkpoint inhibitors 42.

To apply the proposed procedure to clinical medicine, several improvements are required. First, it would be valuable to develop a method for comprehensive phosphoproteomics in even smaller samples. In this study, we utilized 320 μg of protein lysate from three biopsies for each TMT channel. Although the amount of lysate in this study was quite small for phosphoproteome analysis, the need for multiple excisions may be a burden to patients. Thus, further improvements to the current procedure are needed to enable highly sensitive phosphoproteomics from a single biopsy sample containing less than 100 μg of protein. Such a new method with enhanced sensitivity would further allow researchers to investigate the heterogeneity of cancer in terms of the phosphoproteomics.

Another objective for future work is to increase the throughput of the phosphoproteomic analysis pipeline used in this study. Recently, several procedures for high-throughput phosphoproteomics from small amounts of lysate, including clinical tissues, have been reported 43. Integrating these techniques with the protocol proposed here would further expand the utility of phosphoproteomics to other applications, such as large-scale studies to identify diagnostic signatures or screen responses of phosphosignalings to drugs based on phosphoproteomics. Moreover, recent advances in liquid chromatography and mass spectrometry, such as the newly developed technology using ion mobility separation 44, are expected to allow sufficient spectra to be obtained from an even smaller amount of samples. It is expected that combination of these technologies will enable more sensitive global phosphoproteome analysis from small amounts of clinical samples such as endoscopic biopsies.

## Conclusion

In summary, it was demonstrated that global phosphoproteomics could be derived from endoscopic biopsies using a newly developed procedure. This achievement is expected to expand the clinical utility of phosphoproteomics using endoscopic biopsy to further applications including the identification of an optimal treatment strategy. Further studies are needed to reduce the sample loss during the preparation and, thereby, provide deeper phosphoproteomic data. And better decisions about therapeutic targets, especially kinases, would be obtained on the basis of an individual's phosphoproteomic status.

## Supplementary Material

Supplementary figures.

Supplementary table 1.

Supplementary table 2.

Supplementary table 3.

Supplementary table 4.

Supplementary table 5.

Supplementary table 6.

Supplementary table 7.

Supplementary table 8.

## Figures and Tables

**Table 1:** Summary of clinical information on the five subjects for the first phosphoproteomics and on the four subjects for the phosphoproteomics of validation

**Clinical samples for the first phosphoproteomics**							
Patient No	Age	Sex	ECOG PS	Metastatic Site	Histological type	HER2 positivity	Prior chemotherapy
No. 1	73	Female	0	LYM, PER	Intestinal	Negative (IHC 0)	None
No. 2	63	Male	2	LYM, HEP, PER	Intestinal	Positive (IHC 3+)	None
No. 3	79	Male	1	LYM, PER	Diffuse	Negative (IHC 1+)	S-1 plus oxaliplatin
No. 4	78	Male	1	LYM, PER, PUL	Diffuse	Negative (IHC 0)	S-1 monotherapy
No. 5	66	Male	1	LYM	Diffuse	Negative (IHC 0)	S-1 plus cisplatin
							
**Clinical samples for the phosphoproteomics of validation**							
Patient No	Age	Sex	ECOG PS	Metastatic site	Histological type	HER2 positivity	Prior chemotherapy
Val.1	59	Male	1	LYM, HEP, PER	Intestinal	Positive (IHC 3+)	None
Val.2	64	Male	0	LYM, HEP, PER, PUL	Diffuse	Positive (IHC 3+)	None
Val.3	68	Male	1	LYM, HEP, PER	Intestinal	Negative (IHC 0)	None
Val.4	64	Female	0	LYM, PER, PUL	Diffuse	Negative (IHC 0)	None

## Abbreviations

DC: detergent-compatible
DDR: DNA damage response
DTT: dithiothreitol
ECOG PS: eastern cooperative oncology group performance status
FDA: food and drug administration
FDR: false discovery rate
HEP: liver
IAA: iodoacetamide
HRP: horseradish peroxidase
IMAC: immobilized metal affinity chromatography
IHC: immunohistochemistry
KSEA: kinase-substrate enrichment analysis
LC-MS/MS: liquid chromatography-tandem mass spectrometry
LDS: lithium dodecyl sulfate
LYM: lymph node
Lys-C: lysyl endopeptidase
PCA: principle component analysis
PER: pertineum
PTS: phase transfer surfactant
PUL: lung
pY: phosphotyrosine
PVDF: polyvinylidene difluoride
SAM: significance analysis of microarray
SCX: strong cation exchange
SEM: signature enrichment analysis
TMT: tandem mass tag
