# Supplementary material for: Comprehensive characterization of the phosphoproteome of gastric cancer from endoscopic biopsy specimens
Source: Theranostics. 2020 Jan 12;10(5):2115–29. doi: 10.7150/thno.37623 (PMC7019165; doi:10.7150/thno.37623)

Figure S1 A result of the pathway analysis for the first phosphoproteomics based on the pathway information in Wikipathway showing pathways that have p value less than 0.01 representing enrichment in the phosphosites that are increased in cancer tissues relative to normal tissues (red bars) and enrichment in the phosphosites that are decreased in cancer biopsies (blue bars).

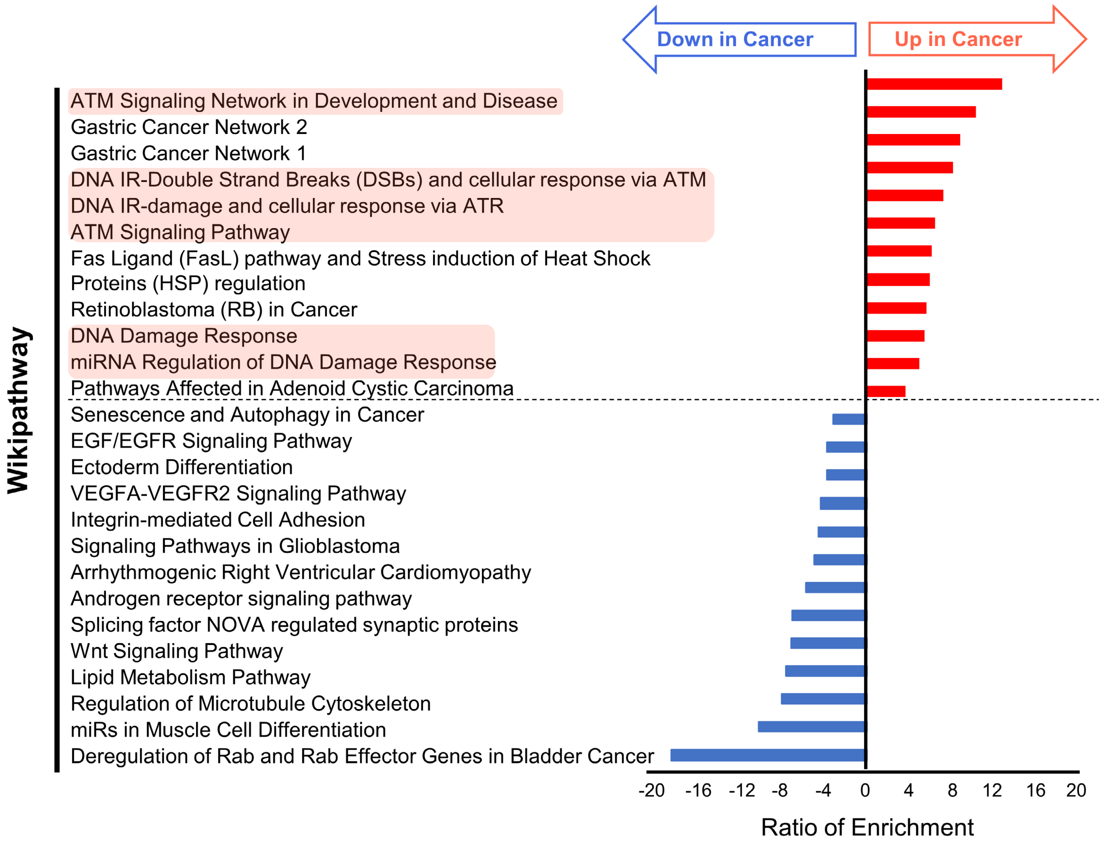

Figure S2 A result of the pathway analysis for the second phosphoproteomics based on the pathway information in KEGG showing pathways that have *p* value less than 0.01 representing enrichment in the phosphosites that are increased in cancer tissues relative to normal tissues (red bars) and enrichment in the phosphosites that are decreased in cancer biopsies (blue bars).

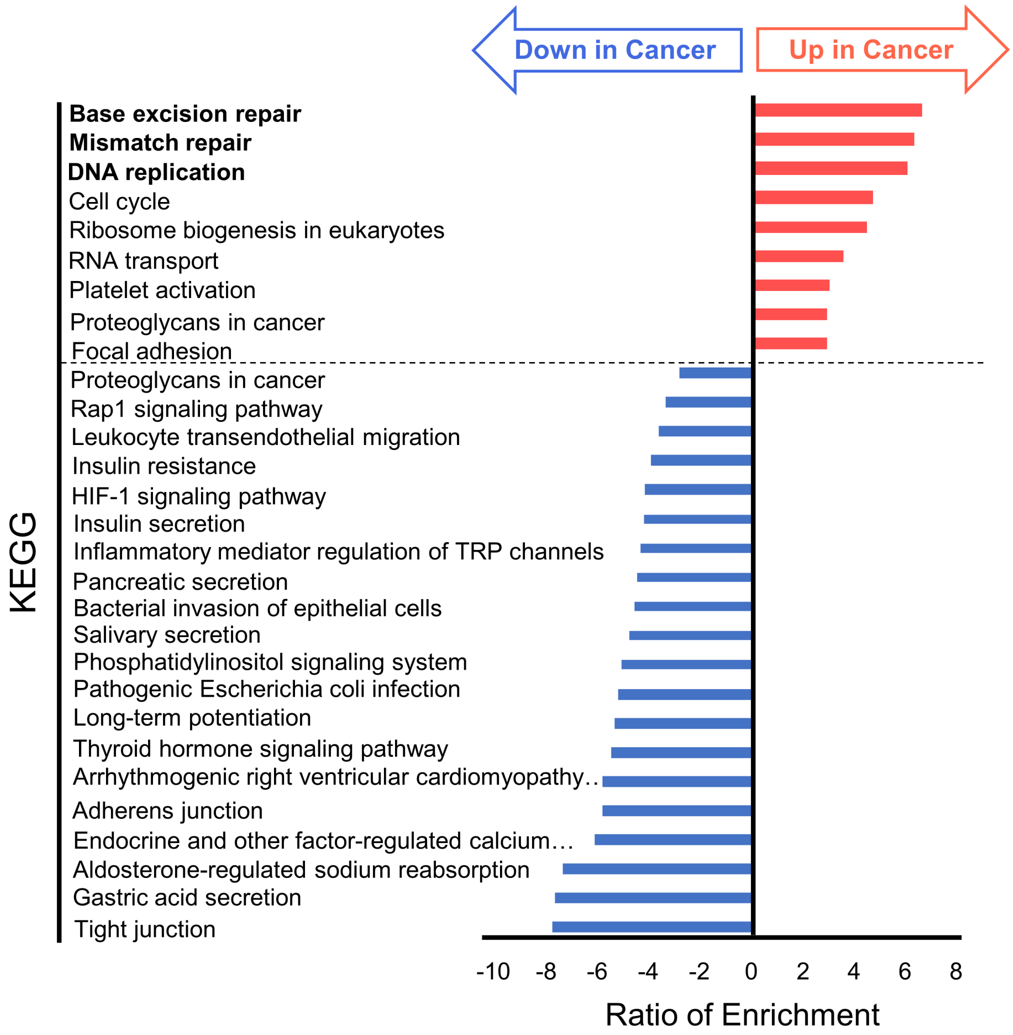

Figure S3 A result of the pathway analysis for the second phosphoproteomics based on the pathway information in Wikipathway showing pathways that have *p* value less than 0.01 representing enrichment in the phosphosites that are increased in cancer tissues relative to normal tissues (red bars) and enrichment in the phosphosites that are decreased in cancer biopsies (blue bars).

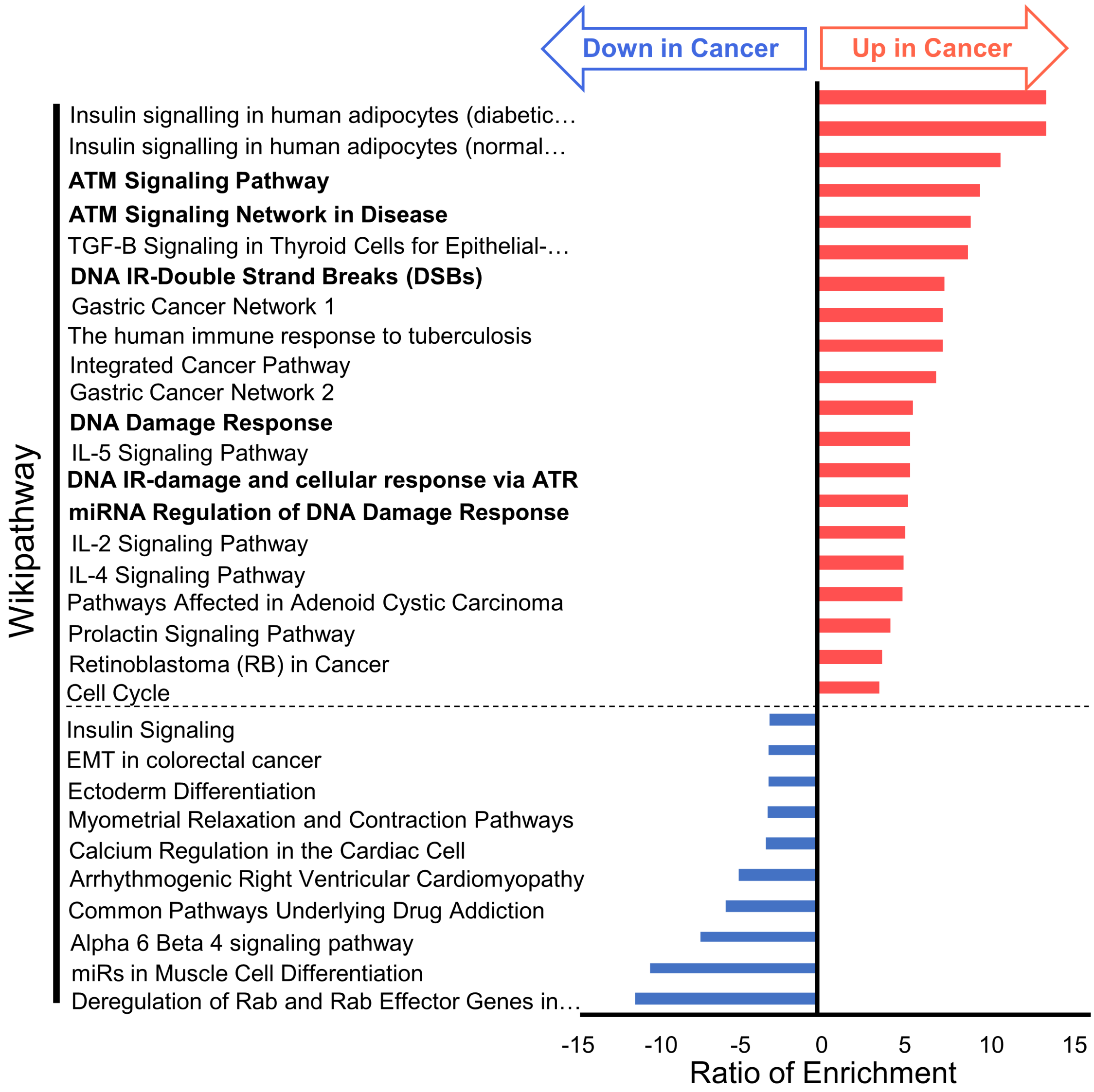

Supplement: Supplementary file 1 — Supplementary figures. [file thnov10p2115s1.pdf]
